# Acute Effects of Nitrate-Rich Beetroot Juice on Blood Pressure, Hemostasis and Vascular Inflammation Markers in Healthy Older Adults: A Randomized, Placebo-Controlled Crossover Study

**DOI:** 10.3390/nu9111270

**Published:** 2017-11-22

**Authors:** Kyle Raubenheimer, Danica Hickey, Michael Leveritt, Robert Fassett, Joaquin Ortiz de Zevallos Munoz, Jason D. Allen, David Briskey, Tony J. Parker, Graham Kerr, Jonathan M. Peake, Natalie M. Pecheniuk, Oliver Neubauer

**Affiliations:** 1 Tissue Repair and Translational Physiology Program, Institute of Health and Biomedical Innovation, Queensland University of Technology, Brisbane, OLD 4059, Australia; kyle.raubenheimer@connect.qut.edu.au (K.R.); danica.hickey@qut.edu.au (D.H.); a.parker@qut.edu.au (T.J.P.); jonathan.peake@qut.edu.au (J.M.P.); 2 School of Biomedical Sciences, Institute of Health and Biomedical Innovation, Queensland University of Technology, Brisbane, OLD 4059, Australia; n.pecheniuk@qut.edu.au; 3 School of Human Movement and Nutrition Sciences, University of Queensland, Brisbane, OLD 4059, Australia; michael.leveritt@uq.edu.au (M.L.); r.fassett@uq.edu.au (R.F.); d.briskey@uq.edu.au (D.B.); 4 Institute of Sport Exercise and Active Living, Victoria University, Melbourne, VIC 8001, Australia; joaquin.ortizdezevallosmunoz@live.vu.edu.au (J.O.d.Z.M.); Jason.Allen@vu.edu.au (J.D.A.); 5 School of Exercise and Nutrition Sciences, Institute of Health and Biomedical Innovation, Queensland University of Technology, Brisbane, OLD 4059, Australia; g.kerr@qut.edu.au

**Keywords:** beetroot juice, dietary nitrate, anti-thrombotic effects, anti-adhesive effects, low-grade inflammation, thrombosis, blood pressure, aging, preserving vascular health

## Abstract

Aging is associated with a vasoconstrictive, pro-coagulant, and pro-inflammatory profile of arteries and a decline in the bioavailability of the endothelium-derived molecule nitric oxide. Dietary nitrate elicits vasodilatory, anti-coagulant and anti-inflammatory effects in younger individuals, but little is known about whether these benefits are evident in older adults. We investigated the effects of 140 mL of nitrate-rich (HI-NI; containing 12.9 mmol nitrate) versus nitrate-depleted beetroot juice (LO-NI; containing ≤0.04 mmol nitrate) on blood pressure, blood coagulation, vascular inflammation markers, plasma nitrate and nitrite before, and 3 h and 6 h after ingestion in healthy older adults (five males, seven females, mean age: 64 years, age range: 57–71 years) in a randomized, placebo-controlled, crossover study. Plasma nitrate and nitrite increased 3 and 6 h after HI-NI ingestion (*p* < 0.05). Systolic, diastolic and mean arterial blood pressure decreased 3 h relative to baseline after HI-NI ingestion only (*p* < 0.05). The number of blood monocyte-platelet aggregates decreased 3 h after HI-NI intake (*p* < 0.05), indicating reduced platelet activation. The number of blood CD11b-expressing granulocytes decreased 3 h following HI-NI beetroot juice intake (*p* < 0.05), suggesting a shift toward an anti-adhesive granulocyte phenotype. Numbers of blood CD14^++^CD16^+^ intermediate monocyte subtypes slightly increased 6 h after HI-NI beetroot juice ingestion (*p* < 0.05), but the clinical implications of this response are currently unclear. These findings provide new evidence for the acute effects of nitrate-rich beetroot juice on circulating immune cells and platelets. Further long-term research is warranted to determine if these effects reduce the risk of developing hypertension and vascular inflammation with aging.

## 1. Introduction

Advancing age is a major risk factor for cardiovascular disease (CVD) [1]. Developing lifestyle-based strategies to prevent adverse changes in arteries with aging has therefore become increasingly important for physiological and biomedical research [1]. Vascular endothelial dysfunction is recognized as a key mechanism underlying cardiovascular pathologies including hypertension; atherosclerosis and thrombosis [1,2]. It develops with aging, and is characterized by several phenotypic changes including alterations toward a vasoconstrictive; pro-inflammatory and pro-coagulant profile [1,3]. The endothelium-derived molecule nitric oxide (NO) has a protective influence on all of these processes. Therefore, the age-associated decline in the production and/or bioavailability of NO plays a central role in the impairment of vascular function [1,2]. Endogenously produced by NO synthases (NOS) including endothelial NOS, NO is an important signaling molecule with various physiological functions in the human body [2,4]. NO relaxes vascular smooth muscle cells; contributes to endothelium-dependent vasodilation; and inhibits platelet aggregation and leukocyte adhesion through the cyclic guanosine monophosphate pathway [4].

There has been an increasing interest in the preventative and therapeutic potential of the nitrate-nitrite-NO pathway as an alternative mechanism of NO production [2,5,6,7]. In this pathway, dietary nitrate is absorbed into the circulation, taken up by the salivary glands, and reduced to nitrite by oral commensal bacteria [5,6,7]. Nitrite in swallowed saliva is then absorbed, and, upon entry into the systemic circulation, further reduced to NO by metalloproteins (e.g., hemoglobin and deoxyhemoglobin), enzymes (e.g., xanthine oxidoreductase) and other compounds with redox potential (e.g., polyphenols) [5,6,7]. Nitrite reduction to NO is accelerated in low oxygen or acidic conditions [5,6,7]. Recent research has suggested that the beneficial cardiovascular health properties of a diet rich in plant foods may be particularly related to the high inorganic nitrate content of vegetables such as green leafy or root vegetables [2,5,8]. A number of studies have shown benefits of dietary nitrate in physiological and clinical settings [9,10]. A meta-analysis by Siervo et al. [8] indicated that inorganic nitrate and beetroot juice supplementation was associated with a significant reduction of blood pressure in adults. Recent studies have also reported beneficial effects of dietary nitrate on the inflammatory and thrombotic profile in young healthy individuals [11], and middle-aged, hypercholesterolemic patients [12]. By contrast, there is only little information available on the potential beneficial health effects of dietary nitrate, and particularly, nitrate-rich foods in older individuals. Dietary supplementation of inorganic nitrate with either beetroot juice [9,13] or sodium nitrate reduced resting blood pressure in healthy older adults [13], lowered daily systolic blood pressure in overweight older individuals [9], and improved several markers of endothelial function in older adults with moderately increased risk for CVD [10]. 

On the contrary, a two-week supplementation period with nitrate-rich beetroot juice had no effect on 24-h ambulatory blood pressure in obese older individuals with type 2 diabetes [14]. Data on whether the effects of dietary nitrate on thrombo-inflammatory biomarkers reported previously in younger adults are also evident in older adults are scarce and insufficient. Counteracting the development of chronic low-grade inflammation and adverse hemostatic changes is considered a central strategy to preserve vascular health with advancing aging [1,3,15]. Hence, there is a clear rationale to examine the potential of dietary nitrate in older adults in this context. 

The aim of this study was therefore to investigate the acute effects of a single dose of nitrate-rich versus nitrate-depleted beetroot juice on blood pressure, biomarkers of vascular inflammation and hemostasis in older adults in the hours after juice consumption. We hypothesized that dietary inorganic nitrate ingestion would reduce blood pressure, and elicit beneficial effects on markers of leukocyte and platelet activation, and hemostasis in healthy older adults.

## 2. Materials and Methods

### 2.1. Study Participants

Seventeen older adults were recruited for this study through advertisements in local newspapers from the general population of greater Brisbane, Queensland, Australia, and through e-mail communication from Queensland University of Technology staff members. All participants provided written, informed consent before their inclusion in the study. Prior to the enrolment into the study, all participants were assessed by a medical physician and classified as clinically healthy. This medical entrance examination included a standard medical history questionnaire, resting electrocardiography, height, weight, body mass index (BMI) and blood pressure measurements. The participants also completed a physical activity questionnaire. Exclusion criteria were as follows: BMI (in kg/m^2^) <18 or >35, any evidence of acute or chronic disease such as severe cardiovascular disease, pulmonary, neural or musculoskeletal disease, osteoporotic fractures and diabetes, acute or chronic pain or disability, smoking, and the use of anti-coagulation, non-steroidal anti-inflammatory, or statin-related drugs. Based on these exclusion criteria, one participant was excluded from the study. Four other participants were unavailable to attend their scheduled trial days and took no further part in the study. The data from another participant were excluded from the data analysis due to difficulty with the blood collections. In total, 12 of the original 17 volunteers completed both study visits. Of these twelve participants (five males and seven females; mean age: 64 years, age range: 57–71 years), one was taking low-dose medication for hypertension (angiotensin converting enzyme inhibitor), one was taking a xanthine oxidase inhibitor, and another one reported using a proton pump inhibitor and a selective serotonin and norepinephrine reuptake inhibitor. The participants’ characteristics are summarized in Table 1. Approval for all experimental procedures was obtained from the University Human Research Ethics Committee at Queensland University of Technology (approval number 1500000881). 

### 2.2. Study Design

The study involved a randomized, double-blind, placebo-controlled, balanced crossover design with a washout period of at least two weeks. After enrollment into the study, the participants were allocated to an intervention plan by block random assignment based on computer-generated random numbers. The interventions consisted of the ingestion of a single dose of either 140 mL of nitrate-rich beetroot juice (HI-NI) or 140 mL of nitrate-depleted beetroot juice as the placebo control (LO-NI). All products were obtained from the same supplier (Beet It, James White Drinks, Ipswich, UK). The nitrate-depleted beetroot juice was generated using a standard ion exchange resin, as described previously [13]. The 140 mL of HI-NI beetroot juice contained 12.9 mmol nitrate (NO^3−^), whereas the 140 mL of LO-NI beetroot juice contained between 0.01 mmol and 0.04 mmol nitrate, according to the manufacturer batch analysis. The two juices were indistinguishable by taste, color, smell and packaging. The investigators involved in the data acquisition and analysis were blind to the trial allocation until the study had been completed and all analyses were performed. 

The participants were provided with a list of high-nitrate foods and instructed to avoid foods on this list for 48 h preceding each of the two trial days. They recorded their food intake for 48 h before the first trial day, firstly to check that they adhered to the dietary protocol, and secondly to replicate this food consumption in the 48 h preceding the second trial day. The participants were also required to refrain from caffeine and alcohol consumption. They were also required to abstain from antibacterial mouthwash and chewing gum in the 24 h preceding each trial, because these might have influences the oral microbiome and nitrate metabolism [6,16]. In the 24 h preceding and throughout the trial period on each trial day, the participants were instructed to avoid any strenuous exercise. 

The participants were required to attend our laboratories at the Institute of Health and Biomedical Innovation, Queensland University of Technology, at Kelvin Crove campus, Brisbane on three occasions. These included the medical entrance examination and the two trial days. On the trial days, all participants arrived at the laboratory in the morning, between 7:00 a.m. and 8:30 a.m., in a rested state, following an overnight fast. Upon arrival at the laboratory, participants were provided with a triaxial-accelerometry-based activity monitor (GENEactiv, Activinsights Ltd., Cambridgeshire, UK) to verify that they maintained low physical levels during the entire trial period. At this time, resting blood pressure was also measured, and a venous blood sample was collected for assessment of a baseline for all measures (PRE). Participants then consumed either HI-NI or LO-NI beetroot juice, as reported above, in addition to a standardized, low nitrate breakfast (Table 2). Resting blood pressure measurements and blood sample collections were then again performed 3 h and 6 h post-ingestion. After the 3 h blood pressure measurement and blood sample collection, the participants were provided another standardized low-nitrate meal. 

### 2.3. Blood Pressure Measurements and Power Density Spectral Analysis

Blood pressure measurements were performed in seated position, in a quiet, light controlled room with an ambient temperature of between 19–21 °C. Blood pressure was measured by light photoplethysmography (Finapres Medical Systems, Ohmeda, Madison, WI, USA). To record systolic blood pressure (SBP), diastolic blood pressure (DBP), and mean arterial pressure (MAP), a photoplethysmographic cuff was placed on the left middle finger. This procedure permitted non-invasive, beat-by-beat blood pressure recordings. Participants were asked to remain in a seated position for a minimum 10 min to stabilize blood pressure. During this time, the finger cuff was fitted and an automatic calibration function was used for a minimum of 3 min prior to ensure accurate blood pressure readings. This calibration function was deactivated during the recording period to prevent signal distortion. Blood pressure signals were recorded at 1 kHz for 12 min at each time point through an analogue-to-digital converter (Powerlab 16/30 model, ADInstruments, Colorado Springs, CO, USA) using commercially available software (LabChart version 7.3.7, ADInstruments, Sydney, Australia). Data were exported from LabChart at 1 Hz. Blood pressure variability arising from neurohumoral regulation mechanisms was assessed by power spectral density (PSD) analysis [17]. Time-based data for SBP were transformed into the frequency domain using the fast Fourier transformation [18]. Total power for the first 512 s of each experimental time point was calculated and expressed as mmHg^2^. The very low frequency (VLF) domain between 0.02 Hz and 0.07 Hz is associated with a myogenic response of the vasculature. The low frequency (LF) domain between 0.07 Hz and 0.15 Hz is linked to the myogenic and sympathetic nervous system response. The high frequency (HF) domain between 0.15 Hz and 0.4 Hz is associated with respiration rate and, possibly, endothelial-derived NO [17]. 

For BP, HR, and PSD analysis, all data were subjected to artifact extraction and filtering following export. For artifact extraction, the mean and standard deviation (SD) were calculated for each individual measurement. Values greater than 2SD on either side of the mean were excluded [19]. Extracted values were replaced through cubic spline interpolation.

### 2.4. Blood Sample Collections

At each sampling time-point, approximately 27 mL of blood was collected through a 21-gauge butterfly needle inserted into an antecubital vein. Blood was collected into vacutainers (BD Biosciences, San Jose, CA, USA), containing either 3.2% buffered sodium citrate (for whole blood hemostasis and flow cytometry analyses), ethylenediamine tetraacetic acid (EDTA; for plasma nitrate analysis), and lithium-heparin (for plasma nitrite analysis). For plasma analyses, blood samples were centrifuged at 1500× *g* for 10 min at 22 °C, within 3 min of collection. Plasma was aliquoted, frozen in liquid nitrogen, and stored at minus 80 °C until analysis.

### 2.5. Plasma Nitrite and Nitrate

Plasma nitrate concentrations were quantified using a kit and calibrators from Cayman chemical (Ann Arbor, MI, USA). All samples were defrosted and subjected to ultrafiltration using a 10–kDa filter from Merk Millipore Ltd. (Tullagreen, Ireland). Of the resulting solution, 40 μL was used for the analysis. Standards were prepared as per the manufacturer instructions. 

Plasma nitrite concentrations were measured (within 30 min of defrosting) by chemiluminescence using Ionics/Sievers nitric oxide analyzer (NOA 280i), as per manufacturer’s instructions (Sievers Instruments, Boulder, CO, USA). Potassium iodide in acetic acid was used as the reductant, which has the potential to convert nitrite to NO but is insufficient to reduce any higher oxides of nitrogen such as nitrate and thus is relatively specific for nitrite. 

### 2.6. Rotational Thrombelastometry

ROTEM (Tem International, Munich, Germany) analysis uses a viscoelastometric method (thrombelastometry) to test hemostasis status in whole blood [20]. Within 4 h of blood sampling, four assays were performed: (1) EXTEM (extrinsically-activated test using tissue factor; assesses the extrinsic coagulation pathway); (2) INTEM (intrinsically-activated test using ellagic acid; assesses the intrinsic coagulation pathway); (3) FIBTEM (extrinsically-activated test using tissue factor and the platelet inhibitor cytochalasin D; assesses fibrin contribution to clot strength); and (4) APTEM (extrinsically-activated test using the fibrinolysis inhibitor aprotinin; assesses hyperfibrinolysis). All assays were performed according to instrument instructions and were performed for a minimum of 30 min at 37 °C. ROTEM variables that were measured included: clotting time (CT, seconds), clot formation time (CFT, seconds), alpha angle (α, degrees), maximum clot firmness (MCF, mm), lysis index at 30 min (LI30), and maximum clot elasticity (MCE). Clotting time provides information concerning the first signs of clot detection and describes how rapidly fibrin formation occurs, and are routinely used in clinical settings to examine coagulation profiles. Clot formation time is the time from clotting time until a fixed level of firmness is reached, and describes the rate of initial clot formation that is mediated by fibrin, activated factor XIII, and thrombin-activated platelets. The α angle is used to determine speed of clot formation. Maximum clot firmness is the maximum clot amplitude achieved prior to fibrinolysis. Lysis index at 30 min is the clot firmness at 30 min after clotting time, and indicates the rate at which fibrinolysis occurred. Maximum clot elasticity is derived from maximum clot firmness (MCF) and indicates the platelet contribution of the clot. Maximum clot elasticity (MCE) was determined by the mathematical formula: MCE = (100 × MCF)/(100 − MCF). To determine how platelets contributed to the clot elasticity, the following mathematical formula was used: MCE_platelet_ = MCE_EXTEM_ − MCE_FIBTEM_. This method has been shown to specifically measure the contribution of platelets to the clot firmness [21]. Quality control was performed using quality-controlled plasma samples with known clotting parameters (ROTROL-N and ROTROL-P) at regular intervals. 

### 2.7. Plasma Hemostasis Analysis

Prothrombin time and activated partial thromboplastin time were measured to provide a clinical indication of the extrinsic and intrinsic coagulation pathways. Analysis of prothrombin time and activated partial thromboplastin time was measured using the KC4 Amelung coagulometer (Trinity Biotech, Bray, Co Wicklow, Ireland). Briefly, prothrombin time was determined by incubating 50 μL of citrated plasma for 3 min before addition of 100 μL of lyophilized thromboplastin and CaCl_2_ to initiate clotting (Neoplastine CI Plus, Stago, Parsippany, NJ, USA). Activated partial thromboplastin time was determined by incubating 50 μL of citrated plasma at 37 °C for 3 min with 50 μL of activated partial thromboplastin reagent (TriniCLOT activated partial thromboplastin, Trinity Biotech, Bray, Co Wicklow, Ireland). Clotting was initiated with 50 μL of 30 mM CaCl_2_ and clotting time was recorded. All measurements were performed in triplicate.

### 2.8. Flow Cytometry

**Figure 1:**
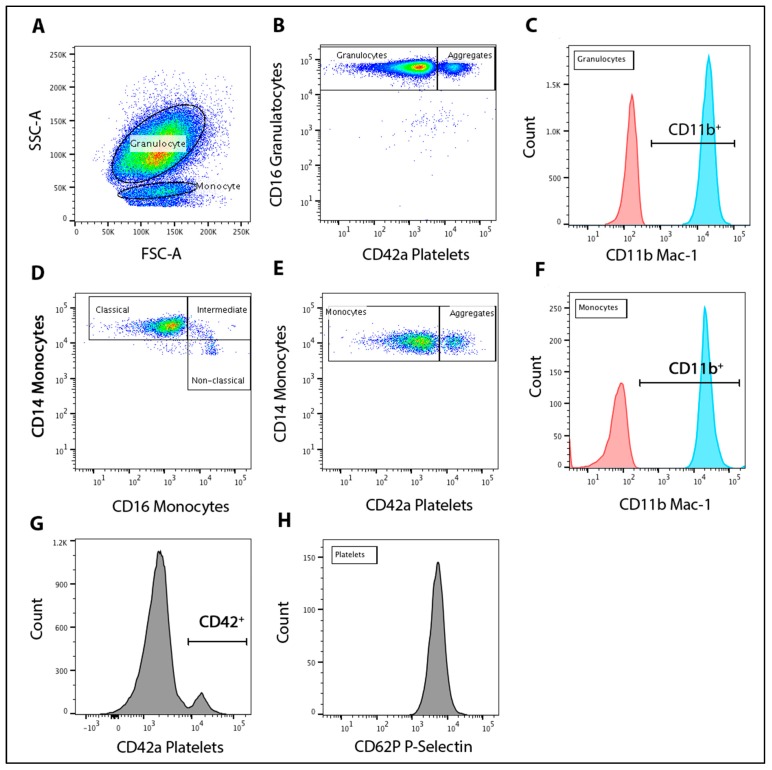
Flow cytometry gating strategy. Granulocyte and monocyte populations gated based on their forward (FSC) vs. side scatter (SSC) properties (**A**). Granulocyte populations were confirmed to be CD16^+^ with granulocyte-platelet aggregates identified by quadrant gating as CD16^+^ and CD42a^+^ cells (**B**) and relative expression of αM integrin expression on granulocytes defined as CD11b^+^ (blue) compared to unstained control (red) via histogram (**C**). Monocyte populations were confirmed to be CD14^+^. Monocytes sub-populations are defined by gating of CD14^++^CD16^−^ (classical), CD14^++^CD16^+^ (intermediate) and CD14^+^CD16^+^ (non-classical) (**D**). Monocyte-platelet aggregates were identified by quadrant gating as CD14^+^ and CD42a^+^ cells (**E**). Relative expression of αM integrin expression on monocytes defined as CD11b^+^ (blue) compared to unstained control (red) via histogram (**F**). Platelet population was identified using histogram analysis of cells CD42a^+^ (**G**) with P-selectin expression on platelets determined through CD62P histogram analysis of CD42a^+^ population (**H**).

Flow cytometric analysis was conducted to measure blood leukocyte-platelet aggregates, blood leukocyte and platelet activation, monocyte population subsets, and platelet P-selectin expression. Monocytes, granulocytes and platelets were immunolabeled in whole blood to prevent artifactual platelet activation that may occur through the isolation of these cells. All monoclonal antibodies were obtained from BD Biosciences. Briefly, 50 μL of whole blood were incubated for 20 min at room temperature with pre-conjugated monoclonal antibodies selective for monocyte-CD14 (Alex fluor 647; AF647), a granulocyte-CD16 (allophycocyanin-conjugated: APC-H7), platelet-CD42a (fluorescein isothiocyanate; FITC), Mac-1/αM-CD11b (phycoerythrin; PE), P-selection-CD62P (Brilliant Violet™; BV421) or isotype matched controls for each fluorochrome (all, BD Biosciences, Eight Mile Plains, Australia). Samples were then fixed and red cells were lysed via the addition of 500 μL of FACS-Lyse solution (BD Biosciences, Eight Mile Plains, Australia). Flow cytometry was conducted using a FACSAria flow cytometer (Becton Dickinson, Franklin Lakes, NJ, USA) with a minimum of 5000 cells for each population collected. Analyses were performed using FlowJo software (Treestar, Inc., San Carlos, CA, USA). The gating strategy for population isolation and relative expression is illustrated in Figure 1.

### 2.9. Leukocyte-Platelet Aggregation and Activation

Granulocytes and monocytes were identified using forward and side scatter characteristics. Granulocytes and monocytes were confirmed, using histogram analysis to be CD16^+^ (CD14^−^) and CD14^+^ respectively. The percentage of granulocyte-platelet aggregates (% GPA) and monocyte-platelet aggregates (% MPA) were determined as the proportion of co-expressing CD16 or CD14 and CD42a platelet marker for each population. Leukocyte activation was measured by the relative expression of CD11b Mac-1/αM marker for each population determined by the median fluorescence intensity (MFI). 

### 2.10. Monocyte Population Subsets

To identify monocyte population subsets, gated CD14^+^ monocytes were plotted against CD16. Monocyte subsets were defined as classical (CD14^++^CD16^−^), intermediate (CD14^++^CD16^+^) and non-classical (CD14^+^CD16^+^) as per previously defined nomenclature [22].

### 2.11. Platelet P-Selectin

Platelet population was identified using a histogram gate to be CD42a^+^ positive. The relative expression of P-selectin (CD62P) in CD42a^+^ platelets was reported as the MFI of CD42a^+^/CD62P^+^ cells.

### 2.12. Statistical Analysis

The sample size was calculated based on previously published data on blood pressure changes following beetroot juice intake in healthy older adults compared with placebo [13]. For detecting a difference with a power of 80% at significance level of α = 0.05, and the conservative assumption of an autocorrelation of 0.9 between repeated measures, a sample size of 13 was required. This figure was inflated by 20% as the estimated dropout rate, resulting in a final sample size of 17. 

GraphPad Prism version 7.00 for Windows (GraphPad Software, La Jolla, CA, USA) was used for statistical analysis. Missing data points were imputed using the expectation-maximum method [23]. Data analysis included baseline comparisons using paired *t*-tests. Two-way repeated measures ANOVA was used to determine the main effects of time, and interaction effects between time and trial. Significant effects of trial and time were analyzed using post hoc tests and a Bonferroni correction. Statistical significance was set at a *p* value of < 0.05. Data are presented as mean ± standard deviation (SD). 

## 3. Results

### 3.1. Plasma Nitrate and Nitrite Levels

**Figure 2:**
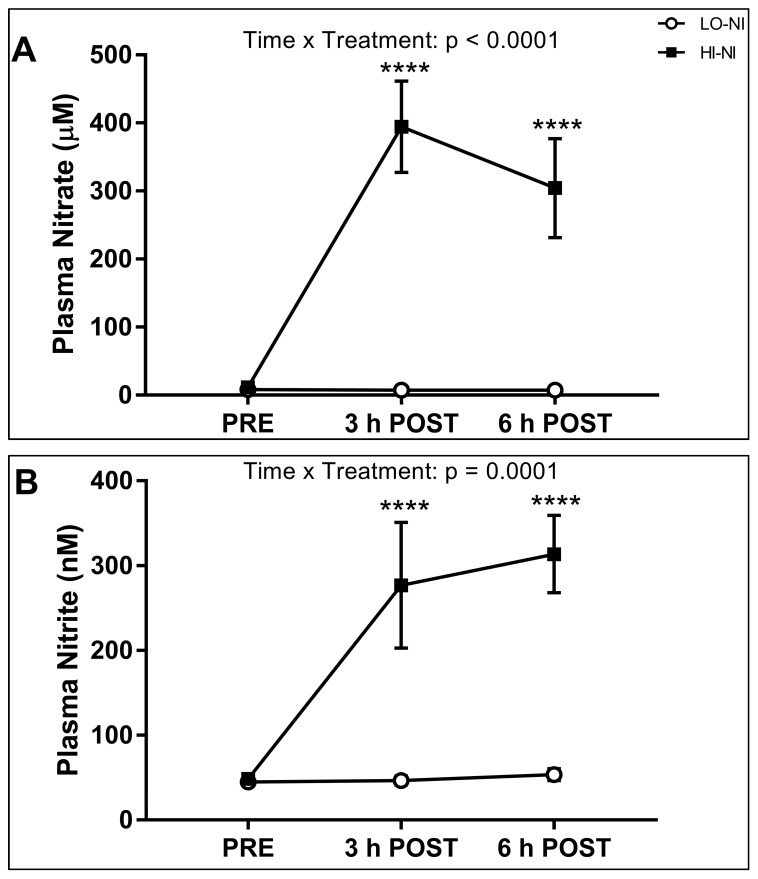
Plasma nitrate (**A**); and nitrite (**B**) concentrations following consumption of nitrate-rich (HI-NI) versus nitrate-depleted (LO-NI) beetroot juice. Data expressed as mean ± standard deviation (SD), *n* = 12. **** *p* < 0.0001, significantly different compared with baseline (PRE). PRE, pre-ingestion (baseline); 3 h POST, 3 h post-ingestion; 6 h POST, 6 h post-ingestion.

Baseline plasma concentrations of nitrate and nitrite were not significantly different in both trials. A time (*p* < 0.05), and a time × trial interaction (*p* < 0.05) were observed for both plasma nitrate and nitrite. Plasma nitrate and nitrite concentration increased at both 3 h and 6 h post-ingestion in the HI-NI trial (*p* < 0.05), but not the LO-NI trial (Figure 2A,B).

### 3.2. Systolic, Diastolic and Mean Arterial Blood Pressure, and Power Spectral Density of Systolic Blood Pressure

**Figure 3:**
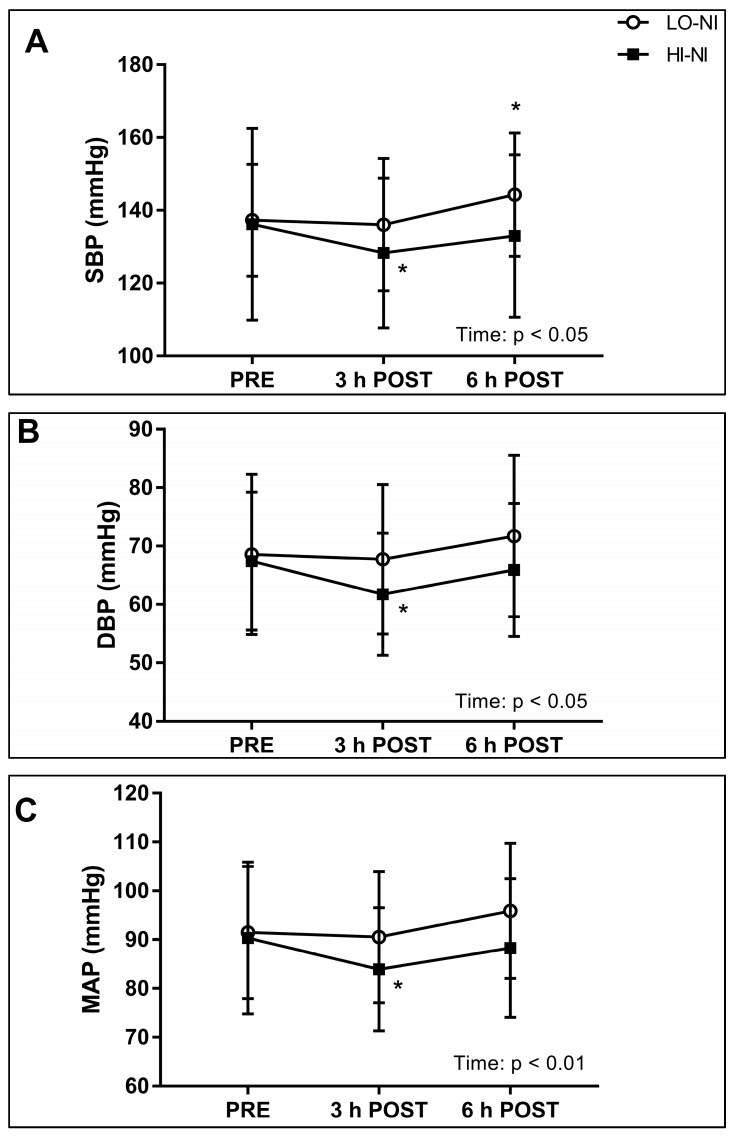
Systolic blood pressure (SBP) (**A**); diastolic blood pressure (DBP) (**B**); and mean arterial pressure (MAP) (**C**) following nitrate-rich (HI-NI) versus nitrate-depleted (LO-NI) beetroot juice ingestions. Data obtained from 12 participants and expressed as mean ± standard deviation (SD). * *p* < 0.05; significantly different changes compared with baseline (PRE): 3 h POST, 3 h post-ingestion; 6 h POST, 6 h post-ingestion.

Baseline levels of SBP, DBP, MAP, and heart rate were not different between trials (Figure 3A–C). A significant effect of time was observed in SBP, DBP and MAP (*p* < 0.05). Post hoc analysis revealed significantly lower values at 3 h post-ingestion as compared to baseline for SBP, DBP, and MAP in the HI-NI trial, but not the LO-NI trial (Figure 3A–C). The mean changes and 95% confidential intervals (CI; reported in brackets) for SBP, DBP, and MAP 3 h after ingestion of the HI-NI beetroot juice were −7.9 mmHg (−1.1 to −14.7 mmHg), −5.7 mmHg (−0.6 to −10.8 mmHg), and −6.4 mmHg (−1.2 to −11.6 mmHg), respectively. In the HI-NI trial, SBP, DBP and MAP had returned to baseline levels at 6 h post-ingestion. A significant increase in SBP between baseline and 6 h post-ingestion was found in the LO-NI trial only. A trend towards a significant effect of time × trial interaction was evident in SBP (*p* = 0.054; Figure 3A). A trend towards time × trial interaction effects was evident in the VLF bandwidth (*p* = 0.06), with an increase in total power for the VLF band observed at 3 h post-ingestion in the HI-NI trial only (Table 3). 

### 3.3. Granulocyte-Platelet and Monocyte-Platelet Aggregation

**Figure 4:**
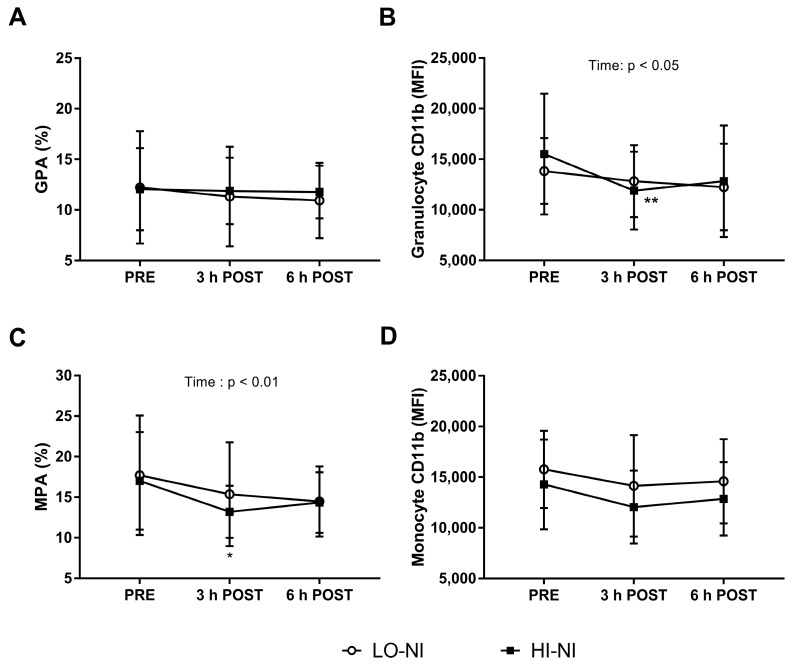
Leukocyte flow cytometry analysis of: blood granulocytes (**A**,**B**); and monocytes (**C**,**D**). Percentage (%): CD16^+^CD14^−^ granulocyte-platelet aggregates (GPA) (**A**); and CD14^+^ monocyte-platelet aggregates (MPA) (**C**). Relative expression of Mac-1/αM integrin (CD11b^+^) on: granulocytes (**B**); and monocytes (**D**), shown as median fluorescence intensity (MFI). Data expressed as mean ± standard deviation (SD), *n* = 12. Two-way ANOVA demonstrated a main effect of time and over the 0, 3 and 6 h time-course for MPA (*p* < 0.05) and granulocyte MFI (*p* < 0.01), with * significant (*p* < 0.05) and ** significant (*p* < 0.01) changes compared to pre-ingestion baseline (PRE) using Bonferroni’s post hoc analysis.

There was no significant difference in GPA (Figure 4A). Whereas no interaction effect was observed between the trials, an overall effect of time on MPA levels in whole blood was observed following both the LO-NI and the HI-NI trial (*p* < 0.01) (Figure 4C). Furthermore, the percentage of MPA significantly decreased (*p* < 0.05) at 3 h post-ingestion compared to baseline following ingestion of HI-NI beetroot juice (Figure 4C). 

### 3.4. Expression of CD11b in Granulocytes with HI-NO Beetroot Juice

The expression of CD11b (Mac-1/αM) on granulocytes (Figure 4B), but not monocytes (Figure 4D) was decreased 3 h after ingestion of HI-NI beetroot juice (*p* < 0.01), as shown by post hoc analysis. For granulocyte CD11b expression, a time-dependent decrease (but no interaction effect) was observed following both the LO-NI and the HI-NO trial (*p* < 0.05) (Figure 4B). 

### 3.5. Monocyte Subset Populations and Platelet P-Selectin Expression

A time × trial interaction effect was evident for intermediate monocytes (*p* < 0.001), with an increase in the intermediate population at 6 h post-ingestion in the HI-NI, and a decrease in the LO-NI group (Table 4). A main effect of time was observed in classical monocytes. No other changes in monocyte subset populations across the assessed time-course were observed. Furthermore, there were no significant main effects, or interaction effects observed for P-selectin in either group. 

### 3.6. Whole Blood Coagulation

There were no significant differences between trials at baseline for any of the ROTEM variables (Table 5). A significant effect of time was evident for INTEM CT (*p* < 0.05). Post hoc analysis showed with a significant decrease of 12.2 s in INTEM CT between baseline and 3 h post-ingestion in the HI-NI trial only (Table 5). There were no significant changes recorded in EXTEM CT, CFT, α angle or INTEM CFT and α angle in either trial. 

There were no effects for EXTEM or INTEM MCF. Analysis of the MCE revealed no significant effects in either trial. A time × trial interaction effect was observed in APTEM CT (*p* < 0.05). Furthermore, post hoc analysis showed a significant decrease in APTEM CT 6 h post-ingestion relative to baseline for the HI-NI only (*p* < 0.05). No significant effects on LI30 were observed for EXTEM or INTEM in either trial. No significant changes were observed for APTEM MCF. 

### 3.7. Effects on Plasma Hemostasis Biomarkers

Clinical coagulation assays were performed on plasma to determine activity of the extrinsic and intrinsic coagulation pathways. Baseline recordings of prothrombin time and activated partial thromboplastin were not different between trials. All recorded prothrombin time and activated partial thromboplastin time values were within reference ranges (Table 5). There was no significant interaction effect, nor any main effects of trial or time for prothrombin time or activated partial thromboplastin time assays (Table 5).

## 4. Discussion

This is the first study to investigate the acute effects of inorganic nitrate-rich versus nitrate-depleted beetroot juice on the functional and phenotypic status of circulating leukocytes and leukocyte-platelet aggregation in older adults. The most important findings were that, 3 h following the consumption of beetroot juice naturally rich in inorganic nitrate, granulocyte and platelet activation were decreased, and blood pressure was also decreased. A small increase was observed in intermediate monocytes 6 h after nitrate-rich beetroot juice ingestion, but it is currently unclear whether this represents an immune activation or pro-inflammatory response. From a practical perspective, these findings are important for the application of beetroot juice (and possibly other nitrate-rich foods) as a nutritional intervention to preserve cardiovascular health with aging. The observed acute anti-thrombotic, anti-adhesive and blood pressure-lowering responses to beetroot juice may accumulate over time, thereby reducing the long-term risk of developing vascular inflammation and hypertension. 

### 4.1. Effects of Nitrate-Rich Beetroot Juice on Plasma Nitrate and Nitrite

Following the ingestion of nitrate-rich beetroot juice, but not the placebo, plasma nitrate concentration increased 3 h post-ingestion, and was still elevated 6 post-ingestion. This indicates that the nitrate in the beetroot juice was absorbed in the gastro-intestinal tract and entered the circulation, resulting in plasma nitrate levels of ≈400 μM 3 h post-ingestion. Comparable plasma nitrate concentrations have been reported in healthy young men after administration of beetroot juice containing 8.4 mmol (plasma nitrate ≈300 μM at 1 and 2 h post-ingestion), and 16.8 mmol nitrate (≈600 μM at 2 h post-ingestion) [24]. Furthermore, we observed an increase in plasma nitrite to ≈300 nM 3 h and 6 h following the ingestion of the nitrate-rich beetroot juice only. This suggests that the circulating nitrate originating from the beetroot juice was taken up by the salivary glands and reduced to nitrite by oral commensal bacteria, and that nitrite in swallowed saliva reached the systemic circulation [5,6,7]. In healthy young men, plasma nitrite concentrations increased to ≈400 nM 2 h after ingestion of 6.8 mmol nitrate, and to ≈650 nM 4 h post-ingestion of 16.8 mmol nitrate [24]. Increases in plasma nitrite have also been reported within three hours after a nitrate-rich meal (≈600 nM) [25] and after short-term beetroot juice supplementation (≈1000 nM) [13] in older individuals. Potential explanations for the differences in the plasma nitrite responses between our study and previous studies in young [24] and older adults [13,25], include a longer supplementation period (2.5 days) [13] versus single intake, inter-individual differences, and effects of aging. Smaller effects of dietary nitrate on plasma nitrite concentrations might be expected in the older population due to age-related changes in oral microbiota and an attenuated enterosalivary circuit [13,25]. 

### 4.2. Effects of Nitrate-Rich Beetroot Juice on Blood Pressure and Frequency Components of Blood Pressure Variability

High blood pressure is a major risk factor for CVD [8,26]. The beneficial effects of the Dietary Approaches to Stop Hypertension (DASH) on blood pressure have partly been attributed to the high inorganic nitrate content of plant foods such as green leafy and root vegetables [5,8]. Although a number of studies have shown blood pressure-lowering effects of dietary nitrate in young to middle-aged individuals [8], our study is among a very few investigations that have involved healthy older adults [9,10,13,14]. Our data showed decreases in mean arterial pressure, systolic and diastolic blood pressure 3 h following the ingestion of nitrate-rich beetroot juice only. A recent study showed decreases in blood pressure in tentatively healthy older individuals following 2.5 day-beetroot juice supplementation [13]. Furthermore, three-week supplementation with beetroot juice reduced daily systolic blood pressure, but not resting clinical blood pressure or 24-h ambulatory blood pressure in older overweight individuals [9]. In contrast, Gilchrist et al. [14] reported no effects of 14 day-beetroot juice supplementation on 24-h ambulatory blood pressure in older individuals with type 2 diabetes mellitus. These variable effects of beetroot juice are potentially due to differences in the participants’ health status. A diminished vascular reactivity and an impaired responsiveness to NO might have attenuated or prevented any effects of beetroot juice on blood pressure in the individuals with type 2 diabetes [14]. Our data support the notion that dietary nitrate is a key component of a fruit and vegetable-rich diet that can potentially help to reduce the risk of developing hypertension with advancing age. Additional research is needed to substantiate these findings and to examine whether these beneficial effects are sustained with long-term dietary nitrate intake. 

By measuring beat-to-beat blood pressure in combination with power spectral analysis (PSD) (Table 3), we aimed to identify individual blood pressure control mechanisms [17]. Blood pressure control involves multiple mechanisms including pressure-volume regulation, the sympathetic nervous system, hormonal regulation such as through the renin-angiotensin-aldosterone-system, and local mechanisms such as the myogenic vascular response and NO [17,26]. The hypotensive effects of dietary nitrate have mainly been attributed to peripheral vasodilation mediated by NO [11]. However, a recent study by Notay et al. [27] showed that high-nitrate beetroot juice intake decreased muscle sympathetic nerve activity at rest and during handgrip exercise in young healthy individuals. This suggests that the mechanisms of action of dietary nitrate involve a neural contribution [27]. Contrary to these results [27], our data suggest no sympathetic modulation of vascular tone by dietary nitrate, as assessed by PSD analysis in the LF range. We also did not observe changes in the respiration-linked HF range. Factors that could have contributed to these differences in the sympathetic nerve responses in the study of Notay et al. [27] and ours include age and different measurement time-points (165–180 min in the study by Notay et al. [27]). We observed a trend towards a difference between the responses to nitrate-rich and nitrate-depleted beetroot juice in the VLF range, with an increase in the VLF range 3 h post-ingestion in the high nitrate trial only. The VLF domain has been linked to the myogenic response of the vasculature [17]. Previous animal data have suggested that NO mediated smooth muscle contraction through Ca^2+^ oscillation [28], whereas a more recent human study showed that dietary nitrate increased skeletal muscle force without changing calcium handling [29]. Whether our data are reflective of an increased myogenic vascular responsiveness needs to be addressed in future studies. 

### 4.3. Effects of Beetroot Juice on Markers of Vascular Inflammation and Platelet Activation

Accumulating data have shown that impaired immune function and low-grade inflammation contribute to the development of major age-related diseases [15,30], particularly including CVD [1]. There is emerging evidence that suggests that dietary nitrate elicits anti-inflammatory and anti-adhesive effects [6]. Previous research in rodents showed that dietary nitrate attenuated the recruitment of leukocytes during acute vascular inflammation [31]. Another study demonstrated inorganic nitrate-induced improvements in the inflammatory status of genetically modified, atherosclerosis-prone mice, including a reduced neutrophil CD11b expression [32]. Thus far, however, only few data are available on the influence of dietary nitrate on the inflammatory and thrombotic profile in humans. Webb et al. reported an inhibitory effect on ex vivo platelet aggregation in plasma that was collected 3 h after beetroot juice ingestion in healthy volunteers [11]. Velmurugan et al. showed that six-week supplementation with nitrate-rich beetroot juice resulted in a reduction in platelet-monocyte aggregates in patients with hypercholesterolemia [12]. In agreement with these available human data, another key finding of this investigation was that nitrate-rich beetroot juice reduced blood granulocytes expressing CD11b at 3 h post-ingestion relative to baseline. CD11b (also known as Mac-1/αM) is a molecule involved in the recruitment of leukocytes to the endothelium. Moreover, we observed a decrease in monocyte-platelet aggregation following nitrate-rich beetroot juice intake only. Monocyte-platelet aggregation plays an important role in the development of inflammation in vascular diseases [33], especially with aging [3], and is considered a reliable biomarker for platelet activation. Notably, significant effects of time were observed in both groups in granulocyte CD11b expression and monocyte-platelet aggregation. This possibly suggests additional effects of bioactive ingredients other than nitrate contained in the beetroot juice and the apple that was part of the standardized breakfast [34]. Beetroot juice in particular contains a variety of potentially bioactive phytochemicals, including betalains and flavonoids, some of which may particularly elicit anti-thrombotic and anti-inflammatory effects [2,35]. 

When further characterizing monocyte population subsets, we observed an increase in circulating intermediate CD14^++^CD16^+^ monocytes 6 h after nitrate-rich beetroot juice ingestion, and a decrease in the placebo trial at the same time-point (Table 4). Intermediate monocytes have previously been related to cardiovascular events in a patient population [36]. However, unlike the healthy participants of our investigation, the patients of this previous study had a high burden of risk factors and cardiovascular disease [36]. The clinical implications of the increase in intermediate monocytes after dietary nitrate intake in healthy individuals are unclear. Additional research is also required to examine whether these changes represents a transient phenotypic shift from classical CD14^++^CD16^−^ to non-classical CD14^+^CD16^++^ monocytes [22]. Together, most of the changes in thrombo-inflammatory markers observed in our study suggest beneficial effects especially of nitrate, but possibly also other bioactive compounds in beetroot juice.

### 4.4. Effects of Nitrate-Rich Beetroot Juice on Hemostasis

In addition to impaired immune function [15], alterations in hemostasis play a critical role in the development of endothelial dysfunction and CVD with aging [3]. Dietary nitrate has been shown to inhibit platelet aggregation in a number of studies in younger individuals [11,12,37]. A recent ex vivo experiment demonstrated inhibitory effects of a NO donor on various blood coagulation processes as measured by increased reaction time of fibrin formation, decreased rate of clot formation and reduced clot strength [38]. Extending upon these studies that focused mainly on the effect of dietary nitrate on platelet activation in younger adults, we investigated various components of coagulation in a population of older adults. Applying thrombelastometry enabled us to comprehensively assess whole blood coagulation [20] (Table 5). Our data showed subtle time-dependent changes in the clotting time via the intrinsic coagulation pathway, as measured by the INTEM test, with a moderate, but significant decrease in INTEM clotting time 3 h after nitrate-rich beetroot ingestion. Furthermore, there was a small decrease in the APTEM clotting time (involving the extrinsic pathway) 6 h after nitrate-rich (but not nitrate-depleted) beetroot juice intake. Notably, the observed changes in clotting times were within clinical reference values. The plasma-based prothrombin time and activated partial thromboplastin time have remained unchanged. It therefore appears unlikely that the observed minor changes in secondary hemostasis are clinically relevant. Together with our findings on platelet-leukocyte interaction, these data suggest that beetroot juice ingestion rather affects the primary hemostasis pathway involving platelet activation and aggregation. 

### 4.5. Theoretical and Practical Implications

We aimed to examine the effects of dietary nitrate in the form of concentrated beetroot juice as a natural foodstuff rather than supplementing nitrate or nitrite salts. It is thought that the consumption of a multitude of phytochemicals as part of a whole foods elicits additive and synergistic effects [2]. The observed significant decreases in granulocyte activation and monocyte-platelet aggregation following both nitrate-rich and nitrite-depleted beetroot juice ingestion may support this concept. Future research may use extracts containing candidate phytochemicals such as specific flavonoids to identify the key bioactive compounds that appear to have contributed to the responses to beetroot juice consumption. Furthermore, as part of a well-controlled study, we collected blood and monitored blood pressure at the same time of the day to avoid circadian effects between the two trials. We cannot exclude the possibility of circadian influences on blood pressure or immunological measurements over the assessed time-period (i.e., from pre- to 6 h post-ingestion). Nor can we completely exclude a possible interference of the medications (other than specified as exclusion criteria), used by some of the participants. Notably though, we observed similar effects of nitrate-rich beetroot juice (such as increases in plasma nitrate and nitrite, and decreases in blood pressure) in the individuals who used medications, as compared with the other participants. We also believe that our study population is representative of what can be considered as clinically healthy older adults.

## 5. Conclusions

These data provide new insights into the acute effects of inorganic nitrate-rich as compared with nitrate-depleted beetroot juice on blood pressure, circulating leukocyte phenotypes, platelet reactivity and whole blood coagulation in a population of older adults. Our data confirm anti-thrombotic and anti-adhesive properties of dietary nitrate that have previously been observed in studies in animals and predominately young humans. Furthermore, our findings support the very few data available on blood pressure-lowering effects of dietary nitrate in healthy older adults. Considering the evidence suggesting anti-inflammatory effects of bioactive plant compounds in the long-term [2,30], it seems very unlikely that the observed increase in intermediate monocytes reflects a shift toward chronic low-grade inflammation. Most of the observed effects can be considered as beneficial for vascular function. Together, our findings provide tentative support of the notion that the regular consumption of beetroot juice could be a key component of lifestyle interventions to preserve cardiovascular health with advancing age. However, long-term studies are warranted to test the benefits of natural beetroot juice and potentially other inorganic nitrate-rich plant foods in this context.

## Figures and Tables

**Table 1:** Characteristics of the study participants.

Anthropometric Characterististics and Medications	Baseline Values
Age (Years; Indicated as Mean Age and Range)	64 (57–71)
Sex (male:female)	5:7
Body mass index (BMI) (kg/m^2^)	25.7 ± 4.2
Baseline systolic blood pressure (mmHg)	133.0 ± 16.6
Baseline diastolic blood pressure (mmHg)	88.6 ± 8.8
Waist circumference (cm)	96.0 ± 12.2
Medications (*n*)	4
ACE inhibitors	1
PPI	1
SSNRI	1
XOI	1

Values are presented as mean ± standard deviation (SD). Abbreviations: ACE, angiotensin converting enzyme; PPI, proton pump inhibitor; SSNRI, selective serotonin and norepinephrine reuptake inhibitor; XOI, xanthine oxidase inhibitor.

**Table 2:** Energy and macronutrient content of standardized low-nitrate meals provided to participants.

**Breakfast**						
**Food**	**Brand**	**Amount**	**Energy (kJ)**	**Fat (g)**	**Carbo-Hydrates (g)**	**Protein (g)**
High-fiber low-sugar cereal biscuit	Weet-bix, Sanitarium	4 biscuits	983	0.9	44.2	8.2
Milk ^†^	Devondale	200 mL	538	6.8	10.2	6.6
Apple Juice	Just Juice	200 mL	374	<1	21	<1
Total			1895	7.7	75.4	14.8
**Snack**						
**Food**	**Brand**	**Amount**	**Energy (kJ)**	**Fat (g)**	**Carbo-Hydrates (g)**	**Protein (g)**
Oat Slice ^ʎ^	Uncle Toby’s	1 bar	600	6.1	19.1	2.1
Apple *	Gala	1 whole fruit	441	0	22.9	0.6
Total			1041	6.1	42	2.7

* Macronutrient data sourced from calorieking.com.au. ^†^ Option as provided for one vegan participant: 200 mL of soy milk (energy = 662 kJ; fat = 9.4 g; carbohydrates = 6 g; protein = 6.2 g). ^ʎ^ Option as provided for one vegan participant: 30 grams of mixed nuts (energy = 765 kJ; fat = 17.5 g; carbohydrates = 2.4 g; protein = 5.0 g). All nutritional values sourced from packaging, unless otherwise stated. Breakfasts were provided immediately following beetroot juice ingestion. Snacks were provided immediately after 3 h assessments.

**Table 3:** Total power spectral density measurements of systolic blood pressure and heart rate following nitrate-depleted (placebo) and nitrate-rich beetroot juice ingestion.

Variable	LO-NI			HI-NI			2-Way ANOVA		
	PRE	3 h POST	6 h POST	PRE	3 h POST	6 h POST	Time	Treatment	Time × Treatment
SBP variability, VLF, mmHg^2^	29.1 ± 4.7	26.6 ± 7.5	28.6 ± 8.1	29.9 ± 5.5	33.0 ± 10.5	27.6 ± 5.1	0.530	0.368	0.065
SBP variability, LF, mmHg^2^	24.7 ± 9.4	25.5 ± 7.1	26.7 ± 8.4	28.3 ± 9.9	31.5 ± 11.3	27.3 ± 8.1	0.578	0.258	0.389
SBP variability, HF, mmHg^2^	24.9 ± 9.2	24.6 ± 5.9	26.4 ± 9.2	26.2 ± 8.2	28.3 ± 11.7	24.5 ± 7.9	0.820	0.740	0.270
Heart rate, beats/min	68.9 ± 4.5	71.3 ± 8.4	69.8 ± 7.8	70.0 ± 6.8	73.7 ± 7.7	71.7 ± 7.4	0.059	0.499	0.857

Data indicated as mean values ± standard deviation (SD), *n* = 12. LO-NI, low-nitrate beetroot juice; HI-NI, high-nitrate beetroot juice; SBP, systolic blood pressure; VLF, very low frequency; LF, low frequency; HF, high frequency; PRE, pre-ingestion (baseline); 3 h POST, 3 h post-ingestion; 6 h POST, 6 h post-ingestion.

**Table 4:** Flow cytometry analysis of monocyte subsets and P-selectin expression following nitrate-depleted (placebo) and nitrate-rich beetroot juice ingestion.

Variable	LO-NI			HI-NI			2-Way ANOVA		
	PRE	3 h POST	6 h POST	PRE	3 h POST	6 h POST	Time	Treatment	Time × Treatment
Classical CD14^++^ CD16^−^ monocytes	90.0 ± 4.1	91.6 ± 2.8	91.2 ± 3.7	91.1 ± 3.6	92.1 ± 3.2	89.7 ± 2.2	0.047	0.991	0.112
Intermediate CD14^++^ CD16^+^ monocytes	4.3 ± 1.5	3.9 ± 1.5	3.3 ± 1.6 *	4.0 ± 1.5	3.5 ± 1.3	4.9 ± 1.0 *	0.183	0.532	0.001
Non-classical CD14^+^ CD16^++^ monocytes	5.4 ± 2.7	4.4 ± 1.4	5.0 ± 2.4	4.2 ± 2.0	4.4 ± 2.1	5.4 ± 1.8	0.235	0.722	0.239
P-selectin (CD42a)	3083 ± 1657	3731 ± 1682	3104 ± 1202	3908 ± 1404	3810 ± 2073	3575 ± 1937	0.385	0.442	0.495

Data indicated as mean values ± standard deviation (SD), *n* = 12. * Significantly different from PRE values, *p* < 0.05; LO-NI, low-nitrate beetroot juice; HI-NI, high-nitrate beetroot juice; PRE, pre-ingestion (baseline); 3 h POST, 3 h post-ingestion; 6 h POST, 6 h post-ingestion.

**Table 5:** Measurement of hemostasis biomarkers following nitrate-depleted (placebo) and nitrate-rich beetroot juice ingestion.

Variable	Reference Range	LO-NI			HI-NI			2-Way ANOVA		
		PRE	3 h POST	6 h POST	PRE	3 h POST	6 h POST	Time	Treatment	Time × Treatment
***Whole Blood Thromobelastometry***										
EXTEM CT, s	42–78	68.9 ± 4.4	71.5 ± 4.4	71.6 ± 5.0	70.3 ± 6.0	68.7 ± 4.7	68.3 ± 7.9	0.904	0.400	0.102
EXTEM CFT, s	53–144	91.8 ± 18.0	93.4 ± 17.2	91.8 ± 14.7	89.8 ± 16.5	94.3 ± 17.7	93.8 ± 21.3	0.271	0.969	0.533
EXTEM MCF, mm	48–70	65.8 ± 4.6	65.8 ± 4.1	66.2 ± 3.6	66.2 ± 3.5	65.3 ± 2.6	65.7 ± 3.7	0.429	0.867	0.495
EXTEM α angle, °	63–83	72.6 ± 3.7	72.4 ± 3.3	72.8 ± 3.2	72.8 ± 3.0	72.1 ± 2.8	72.3 ± 4.0	0.389	0.781	0.304
EXTEM LI30, %	94–100	99.3 ± 0.9	98.8 ± 1.4	99.3 ± 0.8	98.8 ± 2.3	98.3 ± 2.1	99.1 ± 0.8	0.151	0.407	0.904
INTEM CT, s	134–218	208.0 ± 17.9	200.3 ± 15.0	199.0 ± 12.7	205.3 ± 18.5	193.1 ± 14.2 *	197.1 ± 20.8	0.010	0.490	0.698
INTEM CFT, s	52–116	72.6 ± 17.5	66.0 ± 13.5	67.7 ± 11.9	66.8 ± 14.7	66.8 ± 11.9	64.9 ± 12.3	0.180	0.614	0.279
INTEM MCF, mm	47–69	65.4 ± 4.6	65.7 ± 4.2	65.1 ± 3.7	66.3 ± 3.7	65.4 ± 3.3	66.3 ± 3.6	0.814	0.704	0.280
INTEM α angle, °	70–83	75.3 ± 3.5	76.6 ± 2.7	76.1 ± 2.5	76.5 ± 2.8	76.5 ± 2.5	76.8 ± 2.5	0.247	0.562	0.287
INTEM LI30, %	94–100	99.7 ± 0.5	99.3 ± 1.2	99.3 ± 0.8	99.5 ± 0.7	98.4 ± 2.5	99.3 ± 0.8	0.066	0.367	0.378
APTEM CT, s	42–78	66.1 ± 5.4	67.6 ± 4.7	67.7 ± 3.6	68.8 ± 6.9	68.6 ± 5.2	64.8 ± 5.4 *	0.254	0.879	0.043
APTEM MCF, mm	48–70	66.3 ± 4.1	65.9 ± 4.2	65.8 ± 3.3	66.3 ± 4.3	65.3 ± 2.5	66.8 ± 3.7	0.234	0.954	0.232
FIBTEM MCF, mm	7–21	14.9 ± 4.3	14.8 ± 4.6	14.4 ± 4.0	14.5 ± 3.8	14.8 ± 3.8	14.9 ± 3.8	0.943	0.973	0.405
MCE		180.0 ± 37.5	178.7 ± 35.2	178.2 ± 25.8	181.6 ± 28.9	171.8 ± 21.2	176.7 ± 28.7	0.432	0.842	0.614
***Plasma Hemostasis Analysis***										
Prothrombin time, s	11–15	11.9 ± 0.2	11.8 ± 0.2	11.8 ± 0.2	11.9 ± 0.2	12.0 ± 0.2	12.0 ± 0.2	0.851	0.605	0.508
Activated partial thromboplastin time, s	26–37	33.0 ± 1.0	32.5 ± 1.0	32.5 ± 1.0	32.8 ± 1.2	32.5 ± 1.2	32.5 ± 1.1	0.297	0.956	0.912

Data indicated as mean values ± standard deviation (SD), *n* = 12. * Significantly different from PRE values, *p* < 0.05. LO-NI, low-nitrate beetroot juice; HI-NI, high-nitrate beetroot juice; PRE, pre-ingestion (baseline); 3 h POST, 3 h post-ingestion; 6 h POST, 6 h post-ingestion; EXTEM, extrinsically-activated test using tissue factor; assesses the extrinsic coagulation pathway; INTEM, intrinsically-activated test using ellagic acid, assesses the intrinsic coagulation pathway; APTEM, extrinsically-activated test using the fibrinolysis inhibitor aprotinin, assesses hyperfibrinolysis; FIBTEM, extrinsically-activated test using tissue factor and the platelet inhibitor cytochalasin D, assesses fibrin contribution to clot strength; CT, clotting time; CFT, clot formation time; MCF. Maximum clot firmness; LI30, lysis index at 30 min; MCE, maximum clot elasticity.
